# Achieving NPVR ≥ 80% as technical success of high-intensity focused ultrasound ablation for uterine fibroids: a cohort study

**DOI:** 10.1186/s12905-024-03093-0

**Published:** 2024-05-18

**Authors:** Shuang Li, Meijie Yang, Jingwen Yu, Wangwa Ma, Yongbin Deng, Liang Hu, Jin-Yun Chen

**Affiliations:** 1 State Key Laboratory of Ultrasound in Medicine and Engineering, College of Biomedical Engineering, Chongqing Medical University, No.1 Medical College Road, Yuzhong District, Chongqing, Chongqing, 400016 China; 2 College of Medical Informatics, Chongqing Medical University, Chongqing, 400016 China; 3 Chongqing Haifu Hospital, Chongqing, 401121 China; 4 Ultrasound Ablation Center, First Affiliated Hospital of Chongqing Medical University, Chongqing, 400042 China

**Keywords:** High-intensity focused ultrasound (HIFU), Uterine fibroids, Nonperfusion volume ratio (NPVR), Ablation, Re-intervention

## Abstract

**Objective:**

To report the long-term re-intervention of patients with uterine fibroids after ultrasound-guided high-intensity focused ultrasound (USgHIFU) ablation and to analyse the influencing factors of re-intervention in patients in the NPVR ≥ 80% group.

**Materials and methods:**

Patients with a single uterine fibroid who underwent USgHIFU at our hospital from January 2012 to December 2019 were enrolled. The patients were divided into four groups according to different nonperfusion volume ratio (NPVR). Kaplan–Meier survival curve was used to analyse long-term re-intervention in different NPVR groups, and Cox regression was used to analyse the influencing factors of re-intervention in the NPVR ≥ 80% group.

**Main results:**

A total of 1,257 patients were enrolled, of whom 920 were successfully followed up. The median follow-up time was 88 months, and the median NPVR was 85.0%. The cumulative re-intervention rates at 1, 3, 5, 8 and 10 years after USgHIFU were 3.4%, 11.8%, 16.8%, 22.6% and 24.1%, respectively. The 10-year cumulative re-intervention rate was 37.3% in the NPVR < 70% group, 31.0% in the NPVR 70–79% group, 18.2% in the NPVR 80–89% group and 17.8% in the NPVR ≥ 90% group (*P* < 0.05). However, no difference was found between the group of NPVR 80−89% and the group of NPVR ≥ 90% (*P* = 0.499). Age of patients and signal intensity on T_2_-weighted imaging (T_2_WI) of tumours were found to be independent risk factors for long-term re-intervention in the NPVR ≥ 80% group. A younger age and greater signal intensity on T2W images corresponded to a greater risk of re-intervention.

**Conclusion:**

USgHIFU, an alternative treatment for uterine fibroids, has reliable long-term efficacy. NPVR ≥ 80% can be used as a sign of technical success, which can reduce re-intervention rates. However, an important step is to communicate with patients in combination with the age of patients and the signal intensity on T_2_WI of fibroids.

**Trial registration:**

This retrospective study was approved by the ethics committee at our institution (Registration No. HF2023001; Date: 06/04/2023). The Chinese Clinical Trial Registry provided full approval for the study protocol (Registration No. CHiCTR2300074797; Date: 16/08/2023).

**Supplementary Information:**

The online version contains supplementary material available at 10.1186/s12905-024-03093-0.

## Background

Uterine fibroids, also known as leiomyomas, are the most common benign reproductive system tumours in females of childbearing age. The incidence rate of uterine fibroids ranges from 4.5 to 68.6% due to population characteristics such as race, region and health status [1]. Approximately 25% of women with uterine fibroids have clinical symptoms [2, 3], such as menorrhagia, irregular bleeding, pelvic pain, or infertility [4–6], which seriously affect women’s quality of life. Treatment options for uterine fibroids require individualized clinical management that considers both symptom relief and the patient’s desire to pregnancy. High-intensity focused ultrasound (HIFU) ablation could cause instant coagulative necrosis (1–3 s) in a well-circumscribed area of 1.5 × 1.5 × 10 mm through focusing the ultrasound beam on the tumour, including ultrasound-guided high-intensity focused ultrasound (USgHIFU) ablation and magnetic resonance-guided high-intensity focused ultrasound (MRgHIFU) ablation [7]. As a completely noninvasive treatment technology, it damages the target area only and thus is effective, safe and controllable [8].

The nonperfusion volume (NPV) is the volume of the area without perfusion on postoperative contrast-enhanced MR images within three days and was used to evaluate the therapeutic extent. The nonperfusion volume ratio (NPVR), a predictor of re-intervention in the short to medium term, is defined as the proportion of NPV to preoperative fibroid volume [9, 10]. Previous studies showed that the two-year clinical efficacy of USgHIFU was similar to that of myomectomy when the NPVR reached 70% [11]. However, clinical evidence of the long-term efficacy of uterine fibroids after USgHIFU is still lacking. Therefore, the long-term efficacy of USgHIFU and the re-intervention rates at different NPVRs were explored. Furthermore, the influencing factors of long-term re-intervention in patients with NPVR ≥ 80% were analysed to provide a basis for an appropriate therapeutic regimen and case screening.

## Methods

### Patients

Patients with uterine fibroids who underwent USgHIFU at the Minimally Invasive and Noninvasive Treatment Centre of our hospital from January 2012 to December 2019 were enrolled.

The inclusion criteria were as follows: (1) premenopausal women between 18 and 50 years old; (2) patients diagnosed with type 1–6 uterine fibroids by magnetic resonance imaging (MRI); (3) the fibroid diameter was at least 2 cm; (4) patients received fibroid-related treatment for the first time.

The exclusion criteria were as follows: (1) patients with special types of fibroids, such as FIGO type 0, 7 or 8; (2) patients with other gynecological diseases, such as adenomyosis or ovarian tumors; (3) patients with serious organic lesions, such as heart failure or liver cirrhosis; (4) patients diagnosed with or suspected to have a malignant disease, such as sarcoma of uterus; (5) patients unwilling to undergo follow-up.

### USgHIFU ablation

One session of USgHIFU ablation was performed by physicians with at least three years of HIFU clinical experience. A focused ultrasound tumour therapeutic system (Model-JC, Chongqing Haifu Medical Technology Co., Ltd., Chongqing, China) was used. The ultrasound transducer worked with a frequency of 0.5–1.5 MHz, and the energy was adjusted within a range of 350–400 W. The guided ultrasound frequency used for real-time monitoring was 3.5 MHz (Esaote, MyLab70, Italy).

All patients received diet preparation, enema cleansing and skin preparation (shaving, degreasing and degassing) before treatment. Fentanyl (0.8–1 µg/kg) and midazolam hydrochloride (0.02–0.03 mg/kg) were administered to maintain conscious sedation while reducing patient discomfort. The patients were placed prone on the HIFU table with their anterior abdominal wall in contact with degassed water. An adjustable water balloon was placed between the abdominal wall and transducer when necessary in case the bowel blocked the acoustic pathway. The treatment focuses were at least 15 mm from the endometrium and 10 mm from the perimetrium. The ultrasonic sonication time and acoustic power were adjusted based on the change in patient tolerance and the target area. Grayscale changes in real-time ultrasonographic imaging served as the ablation marker. Sonication was terminated when the increased grayscale covered the planned ablation area, and the sonication time was controlled within 3000 s. The patients were instructed to remain in the prone position for two hours after the procedure.

### MRI evaluation and classification

All patients received an MRI scan one week before and within three days after the treatment. T_1_-weighted imaging (T_1_WI), T_2_WI and enhanced T_1_WI were performed with a 3.0-T MRI system (Singa HD Excite, GE Healthcare, USA). The MR images were analysed by a radiologist who had completed five years of specialization in abdominal MR imaging (Reader 1) and validated by another radiologist (Reader 2) who had 15 years of experience in abdominal MR imaging. If there was disagreement, Reader 2 retraced the image, and this was considered the final decision.

The MR images were evaluated and measured as follows: (1) Type of fibroid: types 1–6. The fibroids were categorized according to the Federation International of Gynecology and Obstetrics (FIGO) [12]. (2) Location of the fibroids: anterior or posterior. In the anterior fibroid, the acoustic pathway did not pass through the uterine cavity during USgHIFU ablation. Otherwise, it was a posterior fibroid. (3) Signal intensity on T_2_WI: hypointense, isointense or hyperintense. (4) Enhancement type on T_1_WI: mild, moderate or significant. The degree of enhancement was compared with that of the normal myometrium according to the dynamic contrast-enhanced MR image within 60 s of contrast medium injection. (5) Maximum diameter of fibroids: Three dimensions were measured on T_2_WI before treatment: longitudinal diameter (D_1_), anteroposterior diameter (D_2_) and transverse diameter (D_3_). The maximum of the three dimensions was selected as the maximum diameter of the fibroids. (6) Calculation of fibroid volumes and NPVR: The volume was calculated by using the following equation: *V* = 0.5233 × D_1_ × D_2_ × D_3_; (7) Calculation of the NPVR: The NPV was evaluated according to the formula above for enhanced T_1_WI after treatment. NPVR was defined as NPV/posttreatment fibroid volume × 100%.

### Follow-up

A gynecologist who had been working for more than three years followed up with the patients by telephone according to the follow-up content and criteria set by our research team. If the patients had undergone re-intervention, then the method, as well as the reason and time, needed to be recorded. Surgical treatment due to uterine fibroids was defined as re-intervention, such as myomectomy, USgHIFU, hysterectomy or uterine artery embolization (UAE). Patients who were diagnosed with adenomyosis or malignant disease were also followed up. If patients could not be contacted multiple times within three days, they were confirmed to be lost to follow-up.

### Statistical analysis

SPSS version 26.0 (IBM, Armonk, NY, USA) was used for the statistical analysis. Normally distributed data were reported as the mean ± standard deviation (SD), and nonnormally distributed data were reported as medians and interquartile ranges. Categorical data were expressed as the number and percentage (%). Comparisons among groups were conducted using multiple-factor analysis of variance, the Mann–Whitney U test, the Kruskal–Wallis test, the chi-square test, the continuous correction test and Fisher’s exact test, with a *P* value less than 0.05 considered significant. Kaplan–Meier survival curve was used to explore cumulative re-intervention rates, and Cox regression was used to analyse the influencing factors.

## Results

### Baseline characteristics of patients and long-term efficacy of USgHIFU

**Fig. 1:**
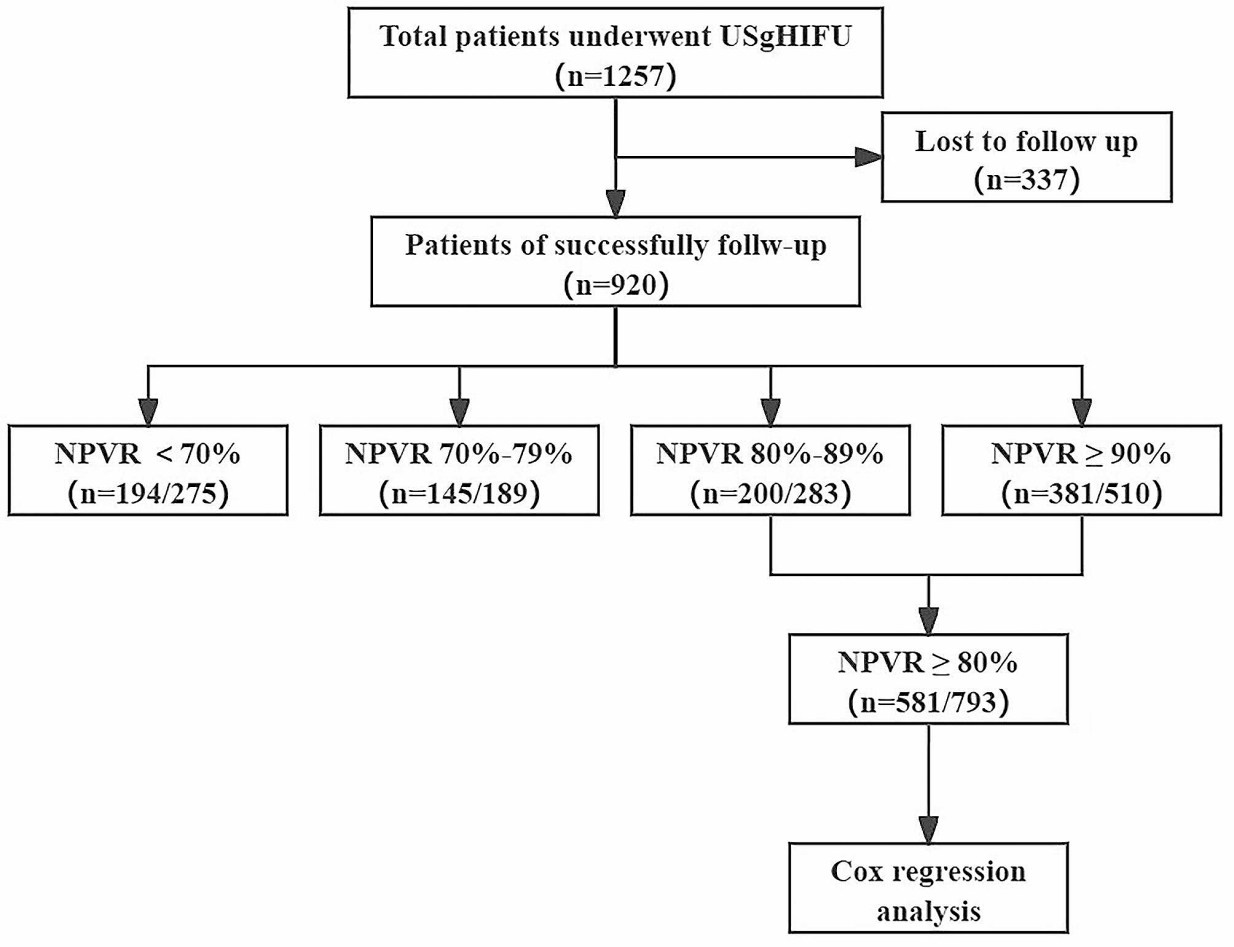
The flowchart of enrollment of patients underwent USgHIFU

As shown in Fig. 1, a total of 1,257 patients were enrolled; 920 patients were successfully followed up, while 337 patients (26.8%) were lost to follow-up. The patients’ median age was 39.0 years (IQR 10.0), and the median BMI was 22.0 kg/m^2^ (IQR 4.0). The maximum diameter of the uterine fibroids was 56.0 mm (IQR 21.0), and the volume of the fibroids was 71.0 cm^3^ (IQR 80.2). Other baseline characteristics are given in Table 1.

**Table 1:** Baseline characteristics of patients and cumulative re-intervention rates

Variable	Total patients (*n* = 1257)	Patients of NPVR ≥ 80% (*n* = 793)
**Number of successful follow-up cases (n)**	920	581
**General patient data**		
Age (years)^*^	39.0 (10.0)	39.0 (10.0)
BMI (kg/m^2^)^*^	22.0 (4.0)	22.0 (4.0)
Family history of fibroids (yes/no) (n)	82/1175	58/735
History of smoking or drinking (yes/no) (n)	76/1181	49/744
History of lower abdominal surgery (yes/no) (n)	427/830	258/525
History of childbirth (yes/no) (n)	920/337	582/211
**Fibroid data of MR imaging**		
Type (I-II/III-IV/V-VI) (n)	143/236/878	92/148/553
Location (anterior/posterior) (n)	860/397	567/226
Signal intensity on T_2_WI (hypointense/isointense/hyperintense) (n)	407/355/495	303/235/255
Enhancement type on T_1_WI of fibroids (mild / moderate / significant) (n)	417/560/280	297/347/149
Maximum diameter (mm)^*^	56.0 (21.0)	56.0 (20.0)
Volume (cm^3^)^*^	71.0 (80.2)	71.3 (78.5)
**Ablation parameters**		
Power (W)	400.0 (0.0)	400.0 (0.0)
Sonication time (s)	880.0 (882.8)	753.0 (702.0)
Dose (KJ)	342.8 (350.0)	291.4 (282.9)
NPVR (%)	85.0 (22.0)	92.0 (10.0)
**Cumulative re-intervention rate (%)**		
1 years	3.4	1.3
3 years	11.8	7.4
5 years	16.8	11.6
8 years	22.6	16.5
10 years	24.1	17.8

Note: ^*^ data was median value and interquartile range in brackets

The median power of USgHIFU ablation was 400.0 W (IQR 0.0), the median sonication time was 880.0 s (IQR 882.8), the median dose was 342.8 KJ (IQR 350.0), and the median NPVR was 85.0% (IQR 22.0%). No SIR C–F complications, such as skin burn or intestinal injury, or long-term complications related to USgHIFU occurred during or after treatment. The median follow-up time after USgHIFU was 88 months (IQR 67, 110), ranging from 45 to 129 months.

**Fig. 2:**
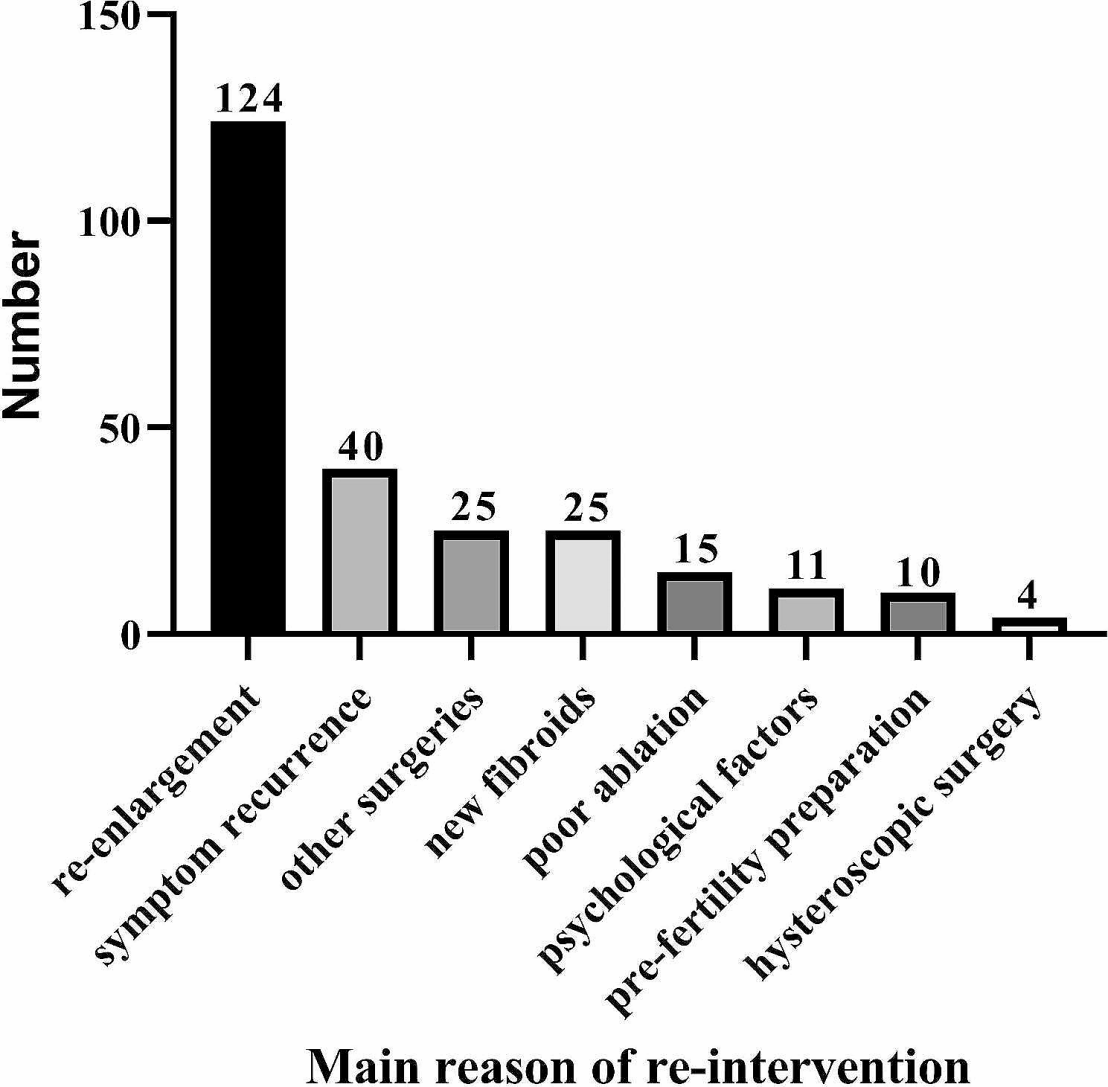
The reasons of re-intervention after USgHIFU

The cumulative re-intervention rates 1, 3, 5, 8, and 10 years after USgHIFU were 3.4%, 11.8%, 16.8%, 22.6% and 24.1%, respectively, as indicated by survival analysis (Table 1). The main reasons for reintervention were re-enlargement of the original fibroids (48.8%, 124/254), followed by symptom recurrence (15.7%, 40/254); removal of the fibroids during other surgeries, such as caesarean Sect. (9.9%, 25/254); new fibroids (9.9%, 25/254); poor ablation (5.9%, 15/254); psychological factors due to the follow-up image of the ablated residual fibroid, which was asymptomatic and did not grow (4.3%, 11/254); pre-fertility preparation (3.9%, 10/254); and hysteroscopic surgery because the submucosal fibroids were expelled (1.6%, 4/254) (Fig. 2). Re-intervention methods included myomectomy (68.5%, 174/254), USgHIFU (17.7%, 45/254), hysterectomy (13.4%, 34/254) and UAE (0.4%, 1/382). In addition, six patients (0.7%) were diagnosed with adenomyosis after USgHIFU, of whom two patients underwent hysterectomy, one patient underwent laparoscopic myomectomy, and the other three patients were temporarily untreated for no obvious symptoms or tolerable dysmenorrhea. Eight patients were diagnosed with thyroid cancer, three patients were diagnosed with breast cancer at the time of follow-up, and all patients underwent surgery without recurrence. In addition, two patients were diagnosed with oophoroma: one patient underwent chemotherapy after surgery, and the other patient died two years ago.

### Cumulative re-intervention in different NPVR groups

**Fig. 3:**
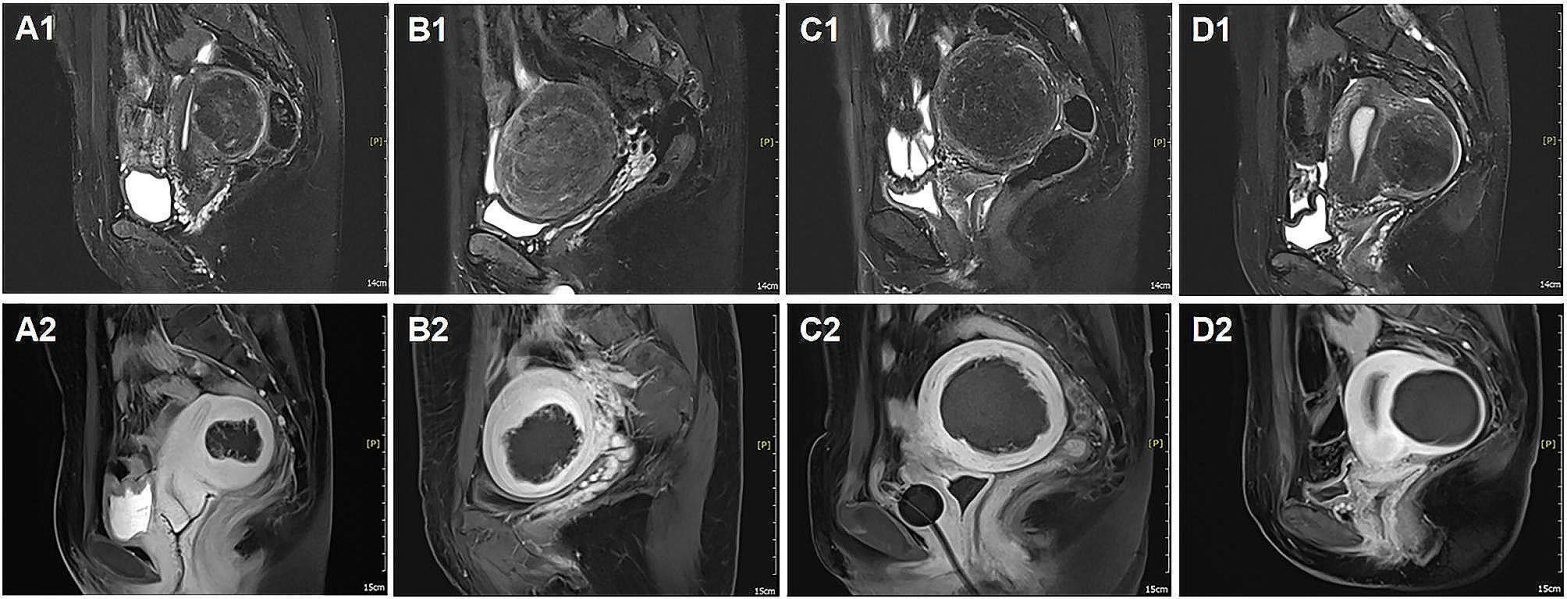
MRI of fibroids with different NPVRs. (**A1**–**D1**) T2-weighted images of fibroids before USgHIFU; (**A**–**D2**) Contrast-enhanced MR image after USgHIFU; (**A2**) NPVR = 30%; (**B2**) NPVR = 73%; (**C2**) NPVR = 88%; (**D2**) NPVR = 100%

**Fig. 4:**
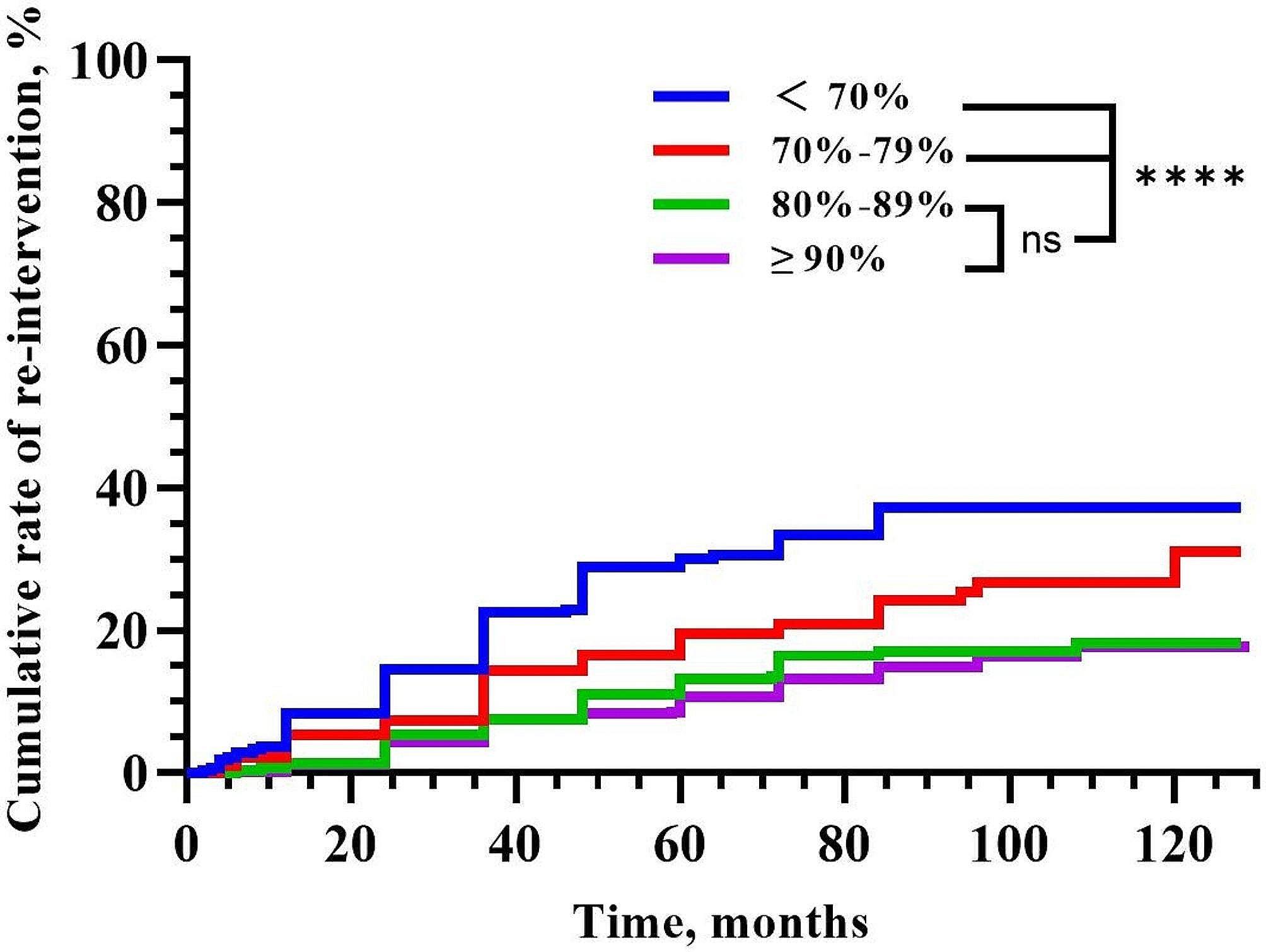
Cumulative re-intervention rates after USgHIFU for different NPVRs according to Kaplan–Meier curve

The patients were divided into four groups according to different NPVRs: < 70% (275/1275), 70–79% (189/1275), 80–89% (283/1275) and ≥ 90% (510/1275) (Fig. 3). The Kaplan–Meier survival curve indicated that the group with NPVR ≥ 90% had the lowest re-intervention rate (17.8%), followed by the group with NPVR 80–89% (18.2%), the group with NPVR 70–79% (31.0%), and the group with NPVR < 70%, which had the highest re-intervention rate (37.3%), with significant differences (*P* < 0.001). However, there was no significant difference in the long-term re-intervention rate between the group of NPVR 80–89% and the group of NPVR ≥ 90% (*P* = 0.499) (Fig. 4).

### Results and influencing factors of re-intervention in patients with NPVR ≥ 80%

On the basis of the above results, 793 patients had an NPVR ≥ 80%, of whom 581 were followed up successfully. The baseline characteristics and cumulative re-intervention rates are shown in Table 1.

Patient characteristics were analysed by Cox regression analysis to determine the influencing factors of re-intervention in patients with NPVR ≥ 80%. The results showed that age, BMI, history of lower abdominal surgery, history of childbirth, fibroid maximum diameter, volume, signal intensity on T_2_WI and enhancement type on T_1_WI were associated with re-intervention, while age and signal intensity on T_2_WI of tumours were found to be independent risk factors. Older patients with a lower signal intensity on T_2_WI of fibroids may have a lower risk of re-intervention (Table 2).

**Table 2:** Cox regression analysis of factors influencing re-intervention in patients with NPVR ≥ 80%

Influencing factors	B	SE	Wald	***P***	Exp(B)	95.0% CI	
						Lower	Upper
Age	-0.096	0.019	24.668	< 0.001	0.909	0.875	0.944
BMI	-0.020	0.036	0.305	0.581	0.980	0.913	1.052
History of lower abdominal surgery	0.357	0.205	3.037	0.081	1.428	0.957	2.133
History of childbirth	0.324	0.269	1.453	0.228	1.383	0.817	2.341
Maximum diameter of fibroids	0.002	0.012	0.041	0.840	1.002	0.979	1.027
Volume of fibroids	0.000	0.000	1.025	0.311	1.000	1.000	1.000
Signal intensity on T_2_WI	0.341	0.131	6.817	0.009	1.407	1.089	1.817
Enhancement type on T_1_WI	0.179	0.142	1.588	0.208	1.196	0.906	1.578

## Discussion

Uterine fibroids, as the most common benign tumours, still have a negative impact on the quality of life of women of childbearing age. With the change in medical concepts, minimally invasive and noninvasive techniques have become the trend of medical development. Myomectomy is a conservative surgical treatment that allows the uterus to be preserved, thus preserving fertility. However, the challenge of minimally invasive techniques such as laparoscopy or robotic-assisted myomectomy arises during the step following specimen excision. In the case of the risk of developing disseminated peritoneal leiomyomatosis or malignancies such as sarcoma, the ExCITE technique was used as an approach for the extraction of myomas [13]. Other minimally invasive treatments, such as radiofrequency myolysolysis [14] and hysteroscopic laser ablation [15], could alleviate symptoms, provide a histological assessment and allow shorter waiting times for spontaneous pregnancy and in vitro fertilization. Additionally, office hysteroscopy is a feasible and highly effective diagnostic and therapeutic procedure that can resolve female infertility related to these pathologies without trauma and with only minimal discomfort [16]. However, USgHIFU, which can immediately cause irreversible coagulation necrosis of the target tissue by using an ultrasound focusing device under the real-time monitoring of ultrasound or magnetic resonance, is a completely noninvasive treatment technology. Patients could walk after USgHIFU without any incisions or bleeding. Moreover, it allows shorter waiting times for a spontaneous pregnancy and in vitro fertilization because no myometrial incisions or sutures are needed. Qin et al. [17] reported that 24 patients experienced unintended pregnancy within one year after surgery, seven of whom delivered and had no serious complications, such as uterine rupture. In recent decades, many studies have shown that USgHIFU is safe and effective in the treatment of uterine fibroids [18]. A meta-analysis revealed that the re-intervention rate was 19.0% at 50 months (range: 17–97) after USgHIFU [19] and 20.7% at 70 months (range: 58–88) [20]. Our study enrolled a total of more than 1,000 patients, which was significantly greater than that in previous single-centre reports. The cumulative re-intervention rate was 16.8% at five years, 22.6% at eight years, and 24.1% at ten years, which was comparable to previous reports. Xu et al. [21] reported that the 5-year re-intervention rate after a myomectomy was 19% according to a meta-analysis. The long-term re-intervention rate of USgHIFU in our study was slightly lower than that of myomectomy.

As an indicator of the success of USgHIFU technology, the NPVR is important for training HIFU ablation technology, clinical treatment plan formulation and efficacy evaluation. A high NPVR reportedly corresponds to a greater reduction in fibroids and increased symptom relief [22, 23]. Park et al. [24] reported that fibroids were reduced by 43% with NPVR > 80% after three months of MRgHIFU, while they were reduced by 20% with NPVR < 80%. The median NPVR of this study was 85.0%. The Kaplan–Meier survival curve for different NPVR groups indicated that a higher NPVR corresponded to a significantly lower re-intervention rate (*P* < 0.001), which was similar to the results of previous studies. The five-year re-intervention rate was less than 15% in the group with NPVR of 80–89% (13.4%) and the group with NPVR ≥ 90% (10.7%), which was lower than that in previous studies. Therefore, the long-term efficacy of USgHIFU is promising in regard to a higher NPVR. Interestingly, the long-term re-intervention rate was similar between the group with NPVR of 80–89% and the group with NPVR ≥ 90%, and the difference was not statistically significant (*P* = 0.880). Liu et al. [25] showed that an NPVR of 70% was considered an appropriate indicator of training quality. Gong et al. [26] showed that experienced doctors can achieve 80% NPVR when treating uterine fibroids, and even more than 90% of doctors have more clinical experience. In addition, some scholars have investigated MRI screening parameters for predicting an NPVR of at least 90% [27, 28]. However, Gong et al. [29] showed that when the NPVR was greater than 90%, SIR-B lower abdominal pain was more likely to occur. The safety and intraoperative response of patients should be considered while pursuing high NPVR during USgHIFU ablation. Therefore, NPVR ≥ 80% can indicate technical success, which means that the NPVR should reach 80% as much as possible under the premise of ensuring safety.

Among the 793 patients with NPVRs ≥ 80%, 17.8% still underwent re-intervention. Cox regression was used to analyse the influencing factors of long-term re-intervention in patients who achieved technical success. Age and signal intensity on T_2_WI are independent risk factors. Older patients and lower signal intensity on T_2_WI of fibroids may indicate a lower risk of re-intervention-. Numerous studies have confirmed that long-term re-intervention is negatively associated with age in UAE, USgHIFU and myomectomy patients [18, 20, 30]. The stimulating effects of oestrogen and progesterone on the growth of fibroids have been confirmed [31]. Most fibroids can shrink or even disappear after menopause without intervention. Fibroid growth and recurrence occur easily in young people, whereas fibroids can be temporarily left without intervention even if they recur in older patients, especially perimenopausal patients. The hyperintense fibroids on T_2_WI revealed sparse collagen fibres and abundant cells [32], which do not easily deposit energy in USgHIFU. Therefore, this type of fibroid tends to have a low reduction rate and a high re-intervention rate [33].

USgHIFU is a noninvasive treatment technique in which the target dissolves, is absorbed or calcified because of the immune system, which means that it cannot disappear immediately after USgHIFU [34]. Approximately 7.5% of patients chose re-intervention only because they had fibroids and did not experience any symptoms. Thus, a necessary step is to fully communicate with patients before treatment to reduce apprehension about USgHIFU ablation.

Eight patients were diagnosed with thyroid cancer, three patients were diagnosed with breast cancer, and two patients were diagnosed with oophoroma at follow-up. Thus, other health problems should also receive attention during health management. According to a large cohort study, the incidence rate of adenomyosis was approximately 0.8% [35]. Moreover, six patients (0.7%) were diagnosed with adenomyosis after USgHIFU, which could indicate that USgHIFU ablation for uterine fibroids does not increase the incidence of adenomyosis.

This paper systematically reviewed eight years of case data, with a maximum follow-up time of up to 129 months. This long follow-up time can confirm the safety and efficacy of USgHIFU. NPVR ≥ 80%, which can be used as a sign of technical success, can provide guidance for clinicians before USgHIFU and serve as a basis for predicting the long-term treatment efficacy of patients after USgHIFU. However, this study is a retrospective analysis with some possible recall bias due to the long time interval. This approach is also limited because many patients did not complete the symptomatic evaluation. Thus, we could not report the symptomatic relief score. More prospective studies are needed to validate this conclusion, and symptoms and quality of life should be assessed during follow-up. A comparative study of myomectomy during the same period is recommended. Furthermore, signal intensity on T_2_WI is one of the influencing factors of re-intervention. T_2_WI hyperintense fibroids had a lower NPVR and greater re-intervention rate than other signal intensities on T_2_WI. How to improve the NPVR and reduce the re-intervention rate of hyperintense fibroids, especially in young patients, is a difficult hotspot issue that needs to be addressed.

## Conclusion

USgHIFU is a safe and efficacious noninvasive treatment. The cumulative re-intervention rates at 1, 3, 5, 8 and 10 years after the procedure were 3.4%, 11.8%, 16.8%, 22.6% and 24.1%, respectively, which are similar to the cumulative re-intervention rates of myomectomy. Moreover, when a higher NPVR is achieved, the re-intervention rate decreases further. NPVR ≥ 80% can be used as a sign of technical success, indicating reliable long-term efficacy and surgical safety. However, age and signal intensity on T_2_WI are independent risk factors for re-intervention even when the NPVR reaches 80%.

In conclusion, USgHIFU, an alternative treatment for uterine fibroids, has reliable long-term efficacy. NPVR ≥ 80% can be used as a sign of technical success, which can reduce re-intervention rates. However, an important step is to communicate with patients in combination with the age of patients and the signal intensity on T_2_WI of the fibroids.

### Electronic supplementary material

Below is the link to the electronic supplementary material.

Supplementary Material 1

## Data Availability

The data that support the findings of this study are available on request from the corresponding author. The data are not publicly available due to their containing information that could compromise the privacy of research participants.
