# Supplementary material for: Achieving NPVR ≥ 80% as technical success of high-intensity focused ultrasound ablation for uterine fibroids: a cohort study
Source: BMC Womens Health. 2024 May 18;24:294. doi: 10.1186/s12905-024-03093-0 (PMC11102242; doi:10.1186/s12905-024-03093-0)
Supplement: Supplementary file 1 — Supplementary Material 1 [file 12905_2024_3093_MOESM1_ESM.docx]

**Supplemental Material**

1. **The interpretation of the fibroid categorization (A1-A4: anterior fibroidsn; B1-B4: posterior fibroids)**

**
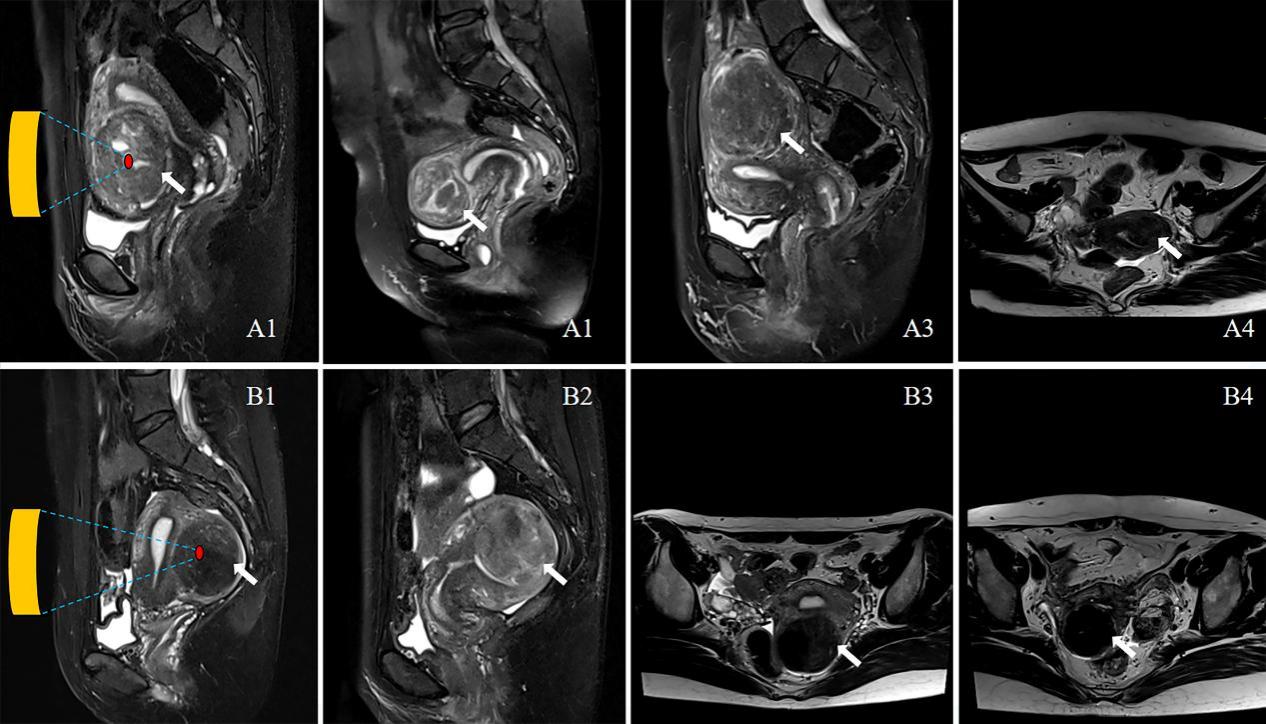
**

1. **The baseline characteristics and ablation results of patients in different NPVR**

| Variable | NPVR＜70%  (n=275) | NPVR 70%-79%  (n=189) | NPVR 80-89%  (n=283) | NPVR≥90%  (n=510) | *P* |
| --- | --- | --- | --- | --- | --- |
| Number of successful follow-up cases (n) | 194 | 145 | 200 | 381 |  |
| General patient data |  |  |  |  |  |
| Age (years)^*^ | 39.0 (9.0) | 39.0 (10.0) | 39.0 (11.0) | 39.0 (10.0) | .951 |
| BMI (kg/m^2^) | 22.0 (3.0) | 22.0 (3.5) | 22.0 (4.0) | 22.0 (4.0) | .448 |
| Family history of fibroids (yes/no) (n) | 12 / 263 | 12 / 177 | 21 / 262 | 37 / 473 | .402 |
| History of smoking or drinking (yes/no) (n) | 17 / 258 | 10 / 179 | 22 / 261 | 27 / 483 | .533 |
| History of lower abdominal surgery (yes/no) (n) | 112 / 163 | 57 / 132 | 88 / 195 | 170 / 340 | .052 |
| History of childbirth (yes/no) (n) | 203 / 72 | 135 / 54 | 208 / 75 | 374 / 136 | .969 |
| Fibroid data of MR imaging |  |  |  |  |  |
| Type (Ⅰ-Ⅱ/Ⅲ-Ⅳ/Ⅴ-Ⅵ) (n) | 30 / 55 / 190 | 21 / 33 / 135 | 28 / 50 / 205 | 64 / 98 / 348 | .878 |
| Location (anterior/posterior) (*n*) | 165 / 110 | 128 / 61 | 202 / 81 | 365 / 145 | .006 |
| Signal intensity on T_2_WI  (hypointense/isointense/hyperintense) (n) | 52/60/163 | 52 / 60 / 77 | 89 / 81 / 113 | 214 / 154 / 142 | ＜0.001 |
| Enhancement type on T_1_WI of primary fibroids  (mild / moderate / significant) (n) | 61 / 129 / 85 | 59 / 84 / 46 | 102 / 123 / 58 | 195 / 224 / 91 | ＜0.001 |
| Maximum diameter (mm) | 56.0 (24. 0) | 58.0 (19.5) | 56.0 (22.0) | 56.0 (21.0) | .330 |
| Volume (cm^3^) | 65.1 (92.9) | 73.5 (81.3) | 69.2 (84.3) | 72.0 (79.7) | .598 |
| Cumulative re-intervention rate (%) |  |  |  |  | ＜0.001 |
| 1 years | 8.4 | 5.3 | 1.4 | 1.2 |  |
| 3 years | 22.5 | 14.3 | 7.4 | 7.5 |  |
| 5 years | 30.1 | 19.6 | 13.4 | 10.7 |  |
| 8 years | 37.3 | 26.7 | 17.0 | 16.3 |  |
| 10 years | 37.3 | 31.0 | 18.2 | 17.8 |  |

Note: Data are median value; interquartile range in brackets.
